# Cognitive support for political partisans’ understanding of policy data

**DOI:** 10.1371/journal.pone.0312088

**Published:** 2024-10-15

**Authors:** Shuyuan Yu, John E. Opfer

**Affiliations:** 1 Department of Cognitive Science, Carleton University, Ottawa, Ontario, Canada; 2 Department of Psychology, The Ohio State University, Columbus, Ohio, United States of America; CNRS: Centre National de la Recherche Scientifique, FRANCE

## Abstract

Political partisanship might lead educated adults–even the highly numerate–to reason selectively about numbers that are relevant to and support their ideology (“motivated numeracy”). In this pre-registered study, we sought to examine the replicability of motivated numeracy, and investigate whether cognitive support (number lines) that improves the reasoning of children might also improve the reasoning of political partisans. To test this, we asked 1000 adults about their political ideology and asked them to interpret fictional data, in a table or number-line format, about ideology relevant (i.e., the effect of gun control on crime) or irrelevant (i.e., the effect of skin cream on rash) issues. We failed to replicate motivated numeracy when political identity was used but observed motivated numeracy when prior attitude was used. Moreover, data presented on number lines elicited 75 percent greater accuracy than data presented in tables, regardless of whether the information was ideology-relevant, or whether data supported, was neutral to, or contradicted participants’ political outlooks. Findings imply that political partisans require cognitive support to be more objective about policy data.

## Introduction

Partisanship can pose several threats to democracy [1]. One threat is that partisan attitudes can distort information processing, making it impossible to reach an evidence-based consensus on policies that would achieve a non-partisan goal. In the United States, for example, the goal of reducing crime is widely shared, yet partisan attitudes exist on whether gun control increases or decreases crime. Similarly, the goal of reducing infectious disease is widely shared, yet partisan attitudes exist on whether mask mandates increase or decrease the number of infections [2]. In theory, an evidence-based consensus could be achieved by addressing these political issues the same way one addresses non-political issues, such as whether a skin cream improves or worsens a rash. Unfortunately, political partisans do not interpret data on the effects of gun control and skin cream with the same objectivity [3]. Rather, attitudes on the potential effects of gun controls, mask mandates, immigration controls, fiscal spending controls, and many other issues, are aligned along partisan dimensions, thereby creating an ‘us’ versus ‘them’ heuristic.

The reason might be due to “motivated reasoning” [4, 5], wherein individuals selectively use their analytical skills to reinforce their own valued views when evaluating new information [6, 7]. This phenomenon results in highly educated and scientifically literate individuals exhibiting increased polarization [8, 9].

Partisan differences might be reduced by promoting numeracy skills, the ability to reason with quantitative information [10, 11]. By knowing how to turn questions about gun control, mask mandates, or skin creams into a mathematical problem with an objectively correct answer, numerate citizens could independently reach a shared conclusion without the need for partisan prodding. If this view were correct, the highly numerate would be expected to exhibit fewer political differences than others when facing the same evidence. By improving numeracy, math education would be expected to facilitate an evidence-based consensus and improve democratic governance.

Numeracy skills, however, may increase polarization instead. Some research suggests that partisans selectively use their numeracy skills to interpret data in a way that just confirms their pre-existing, partisan beliefs. As a result, numeracy skills may actually increase partisan differences on an issue, a phenomenon known as “motivated numeracy” [3, 8]. An implication of this view is that improving numeracy would just increase polarization, as partisans simply pick up a more powerful weapon to support their cause. In other words, motivated numeracy suggests that individuals with better numeracy skills would demonstrate increased, rather than decreased, motivated reasoning.

In this study, we sought to explore whether supports that help children’s quantitative reasoning (number lines) might also help political partisans reach an evidence-based consensus. Specifically, we conducted a pre-registered study to investigate what format of data can reduce subjective biases in interpreting objective numerical data. Below we will review current research on motivated numeracy, the specific challenges of reasoning with rational numbers, and how number lines can be a cognitive tool to support numerical objectivity.

### Motivated numeracy

Generally, numeracy skills are important for personal success. Positive financial and health-promoting behaviors are associated with higher numeracy skills, even after controlling for general intelligence [12–15]. This relation makes sense because understanding financial and health statistics requires the ability to understand rational numbers, such as percentages, ratios, frequencies, and probabilities.

In reasoning about political issues, however, higher numeracy skills may not be as successful. Rather, individuals might be motivated to use their numeracy skills to interpret data to confirm their political ideology or pre-existing beliefs, i.e., “motivated numeracy” [3, 8, 16].

To illustrate, consider this fictional data: “Among gun-controlling cities, 223 of them increased crimes and 75 of them decreased crimes. Among non-gun-controlling cities, 107 of them increased crimes and 21 of them decreased crimes.” Based on this data, does gun control increase or decrease crime? To solve the problem, one must compare the fraction 223/(223+75) to the fraction 107/(107+21). Correctly interpreting the data requires relational reasoning, which is more cognitively demanding and time-consuming than the well-practiced whole number manipulation [17–23]. A common error is to focus only on the numerators and compare the number of crimes increasing among gun-controlling and non-gun-controlling cities (223 vs. 107), thus mistakenly concluding that gun control increased crimes. This mistake would be worse than ignoring the numbers and guessing randomly, as one might expect from someone totally innumerate.

**Fig 1:**
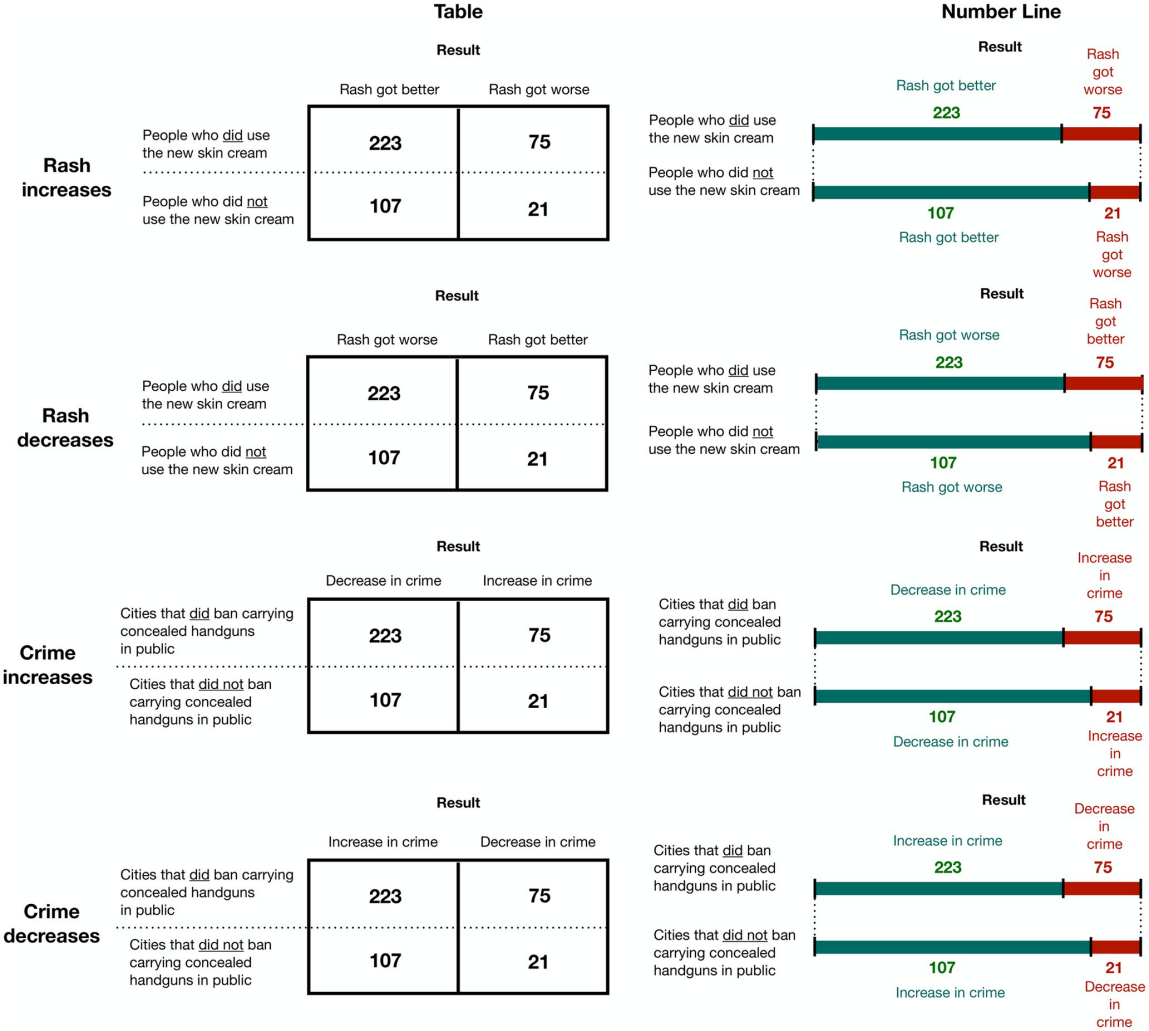
An illustration of the covariance task across conditions and formats. Participants were randomly assigned to one of the 4 conditions (rash increases, rash decreases, crime increases, and crime decreases) and solved the problem in both formats (table, line) for each condition. The order of the format is counterbalanced across participants. The labels of conditions on the left indicate the true interpretation of data.

This fictional data was presented by Kahan and colleagues (2017) to a large, nationally diverse sample [3]. The data was presented in a two-by-two contingency table (Fig 1, bottom left), and participants were randomly assigned either a version in which gun control increased crime or a version in which gun control decreased crime. Consistent with motivated numeracy, participants with higher numeracy skills were more likely to correctly interpret the data when the correct interpretation of data was consistent with their political identity. For example, liberals were more likely to correctly interpret the data when the data showed that gun control decreases crime, whereas conservatives were more likely to correctly interpret the data when the data showed that gun control increases crime. When the information disconfirmed participants’ political ideology, people with higher numeracy skills performed no better than those with lower numeracy skills. In contrast, when the background scenario of the same data is partisanship-irrelevant (e.g., whether a new skin cream reduces rashes), individuals with high numeracy skills were more accurate regardless of their ideology, suggesting that people selectively use their numeracy to support their pre-existing beliefs.

Supporting this view, numerous studies have generalized the concept of “motivated numeracy” to a wide range of political topics [24] and across diverse cultural contexts [25]. However, there is also some contrasting evidence indicating that high numeracy individuals do not necessarily become more polarized [26–28]. In fact, there is even evidence suggesting that individuals with greater numeracy skills might exhibit reduced bias about politically charged topics [29–32]. Furthermore, a direct replication of the original study [3] has yielded inconsistent findings [33], as demonstrated by a recent large-scale pre-registered study. In light of these divergent findings, the first objective of the current research is to investigate the replicability of “motivated numeracy” by conducting a pre-registered study.

### Number line as a tool in teaching rational numbers

The second objective is to explore potential strategies to facilitate comprehension and interpretation of data involving rational numbers. A key obstacle in correctly interpreting a two-by-two contingency table may stem from difficulties in dealing with fractions. To reach correct solutions, participants need to compare two proportions of positive to negative outcomes, rather than fixating on absolute numbers. This challenge is closely associated with the well-documented phenomenon in the field of developmental math cognition known as the “whole number bias” [34–36], describing the tendency to be lured by the integer components of fractions. For example, in fraction comparison, students tend to mistakenly think 4/12 is larger than 2/3 because the integer components (i.e., numerators or denominators) are larger [37]. Even after years of instruction, adults continue to make whole number bias errors [38–41], even including math experts [42].

Research has shown that well-designed visual aids, such as icon arrays, bar and line charts, and others, promote rational number reasoning in various medical and educational contexts [43–46]. A recent systematic review of publications involving more than 15,000 diverse participants from 60 counties summarized that visual aids improve interpreting health risk data by encouraging thorough deliberation, enhancing cognitive self-assessment, and reducing conceptual biases in memory [47]. Moreover, visual aids have been found to be particularly beneficial for individuals with low numeracy skills.

Although not commonly used in health statistics and science communication, in the field of math cognition, number lines are a particularly helpful visual aid for reasoning about ratios. Number lines can be especially effective in communicating ratio information because it reduces a two-part relation to a single one-dimensional value. Previous studies have shown that presenting fractions on number lines facilitates fraction understanding [48–54]. Compared to area models, which emphasize part-whole relationships, the unidimensionality of number lines highlights both the holistic value of fractions [50] and their use in measurement [55]. Fraction number line interventions have been shown to improve understanding of fractions among typically-achieving students and at-risk learners [56–58].

Depicting fractions on number lines also helps adults reason about health-related math problems. Compared to a two-by-two contingency table illustrating the fatal rate of COVID-19 vs. the flu, data presented in number line format reduced the misinterpretation that COVID-19 was less fatal than the common flu [21, 23, 59]. Furthermore, participants randomly assigned to the number-line visual display condition were more accurate with solving health-related math problems than risk-ladder or icon-array visual display conditions, which did not differ from the no-visual-display-at-all condition [60], suggesting that the effect of number line presentation is not merely due to visual presentation of data. Other research showed that horizontal bar charts are as effective as, or more effective than icon arrays [61, 62] in understanding health risk data and reducing bias. However, number lines have not been commonly used in the context of policy data presentation. It remains an open question of whether number lines yield more accurate data interpretation than the table in politically charged items among political partisans.

### Current research

In the current research, we aimed to address two primary research questions. First, we sought to replicate the phenomenon of “motivated numeracy”. Second, we aimed to investigate whether the support that helps children’s quantitative reasoning (number lines) might also help political partisans.

To accomplish these objectives, we presented 1000 adults with a covariance task either on partisanship-relevant or irrelevant topics, presented in a table format and a number line format. Our study design and data analyses were pre-registered. We had five pre-registered hypotheses:

Hypothesis 1: If number-lines improve accuracy, then the mean accuracy of data presented in the number-line format will be higher than that of the table format.
Hypothesis 2: If number-lines improve accuracy by reducing bias, then there will be an interaction between problem type and format (number lines help more on political problems than non-political problems).
Hypothesis 3: If numeracy amplifies political bias (“motivated numeracy”), then there will be a 3-way interaction among problem type, political outlook, and numeracy.
Hypothesis 4: If number-lines improve accuracy by reducing motivated numeracy (“cognitive support for objective numeracy”), then there will be a 4-way interaction between problem type, format, political outlook, and numeracy: numeracy amplifies political bias for the table, numeracy reduces political bias for number line.
Hypothesis 5: If abilities to solve the covariance task in the number-lines format transfer to the table format, then there will be an interaction between trial order and format: accuracy for solving the table problem after the line problem was greater than solving the table problems first.

## Methods

The study was preregistered at https://osf.io/5nkdm after collecting and analyzing partial data (n = 429). All study materials and data can be found at https://osf.io/7486j/. This study was approved by the Institutional Review Board (IRB) of the Ohio State University (Project 2019B0036: Development of Numerical Concepts and Mathematical Skills). Informed written consent was obtained from all participants. Data was collected from March 11th, 2021 to December 9th, 2021.

### Participants

One thousand adult US citizens (*M* = 39.49, *SD* = 11.42, 40% females, 78% white, 7% Asian, 10% African American) recruited from Mechanical Turk participated in the study. Twenty-one percent self-identified as Republican, 26% as Independent, and 53% as Democrats; 33% identified themselves as ‘Very Conservative’ or ‘Conservative’, 18% as ‘Moderate’, and 49% as ‘Very Liberal’ or ‘Liberal’. Participants’ mean educational level was ‘Bachelor’s degree’, and mean annual income was $50,000-$59,999. Participants received a small monetary reward (i.e., $1) for their participation.

### Procedure

Participants completed all measures online through Qualtrics. First, they filled in demographic information (e.g., date of birth, race, and gender). Then they completed the prior attitude measure. After that, they were randomly assigned to one of four Covariance tasks (i.e., rash increases, rash decreases, crime increases, and crime decreases), and each participant completed the assigned task in both table and line format sequentially. The order of format was counterbalanced among participants. Finally, they completed the numeracy scale followed by the political outlook scale. Finishing the procedure took approximately 10 minutes.

### Measures

#### Covariance task (First trial)

The covariance task was adapted from Kahan et al. (2017) [3]. In this task, participants were asked to make causal inferences from fictional data in politically controversial situations (i.e., whether banning concealed guns would increase or decrease crime), or politically neutral situations (i.e., whether a new cream would increase or decrease rash). To investigate the effect of the information presentation format, we randomly assigned participants to one of the 8 conditions: 2 (format: table, number-line) * 4 (problem type: rash increases, rash decreases, crime increases, and crime decreases).

For the politically controversial situation (i.e., crime increases or crime decreases), participants were presented with a paragraph saying that “City government was trying to decide whether to pass a law banning private citizens from carrying concealed handguns in public. Government officials were unsure whether the law will be more likely to decrease crime by reducing the number of people carrying weapons or increase crime by making it harder for law-abiding citizens to defend themselves from violent criminals. As a result, it is necessary to study the effect of the law to see whether it decreases or increases crime.

Researchers have conducted a study. In the study, one group of cities recently enacted bans on concealed weapons, and a second group of cities had no such bans.

In each group, the number of cities that experienced decreases in crime and those that experienced increases in crime in the next year are recorded below. The total number of cities in each two groups is not exactly the same, but this does not prevent assessment of the results.”

Following the paragraph, participants in the table condition saw the two-by-two covariance table, depicting the number of cities that did (or did not) ban control and experienced decreases (or increases) in crime (as in Khan et al, 2017; see Fig 1, bottom left). Participants in the line condition saw the same texts and numbers, except that numbers were depicted in vertically aligned number lines with the same length. On each line, the number of decreases was shaded in green, and the number of increases was shaded in red (Fig 1, bottom right).

Participants were randomly assigned to the data table whose true interpretation states that cities that enacted gun control were more likely to increase or decrease crimes. To control for the perceptual information, the true interpretation of data (crime increases or crime decreases) was manipulated by switching the column labels.

After the data, participants were asked to “indicate whether the study shows that a ban on carrying concealed handguns is likely to make crimes decrease or increase.” They needed to choose between two options, “A) Cities that enacted a ban on carrying concealed handguns were more likely to have a decrease in crime than cities without bans, and B) Cities that enacted a ban on carrying concealed handguns were more likely to have an increase in crime than cities without bans.” The outcome variable was the accuracy of solving the problem, i.e., correctly interpreting the data (=1).

The structure of the politically neutral questions (i.e., rash increases and rash decreases, see Fig 1 top panels) was the same as the politically controversial questions, except that the background was about whether a new skin cream can treat rash.

This task was designed to be difficult in nature. The top left data table in Fig 1 provides several ‘garden paths’. If participants look only at those who used the skin cream (i.e., the first row) and compare the number of people whose rash got better to the number of people whose rash got worse, participants would likely conclude that the new skin cream caused the rash to get better. If participants look only at those who got better (i.e., the first column) and compare the number of people who did use the new skin cream to the number who did not use the new skin cream, participants would also likely conclude that the new skin cream causes the rash to get better. To avoid these garden paths, participants must compare two ratios: the ratio of rash getting better to rash getting worse among those who did use the new skin cream, and the ratio of rash getting better to rash getting worse among those who did not use the new skin cream. The overall mean accuracy was 49.90% (*SD* = .50), which is very close to the expected accuracy from random guessing.

#### Covariance task (Second trial)

To explore whether learning from one format of data presentation might transfer to another format, participants completed a second trial of the covariance task. The second trial was the same as the first trial except that data was presented in the opposite format (e.g., if a participant sees a number-line format on the first trial, they will see a table format of the same scenario on the second trial). Again, participants needed to choose between two opposite interpretations of data, and the outcome variable was the accuracy of solving the problem, i.e., correctly interpreting the data (=1). Mean accuracy was 51.60% (*SD* = .50). The aim of including the second trial was to explore the transfer effect of seeing number line format before the table format. However, strong evidence of transfer would have required four groups (line/line, line/table, table/line, table/table) to assess the independent effect of the first trial on the second trial.

#### Prior attitude

**Fig 2:**
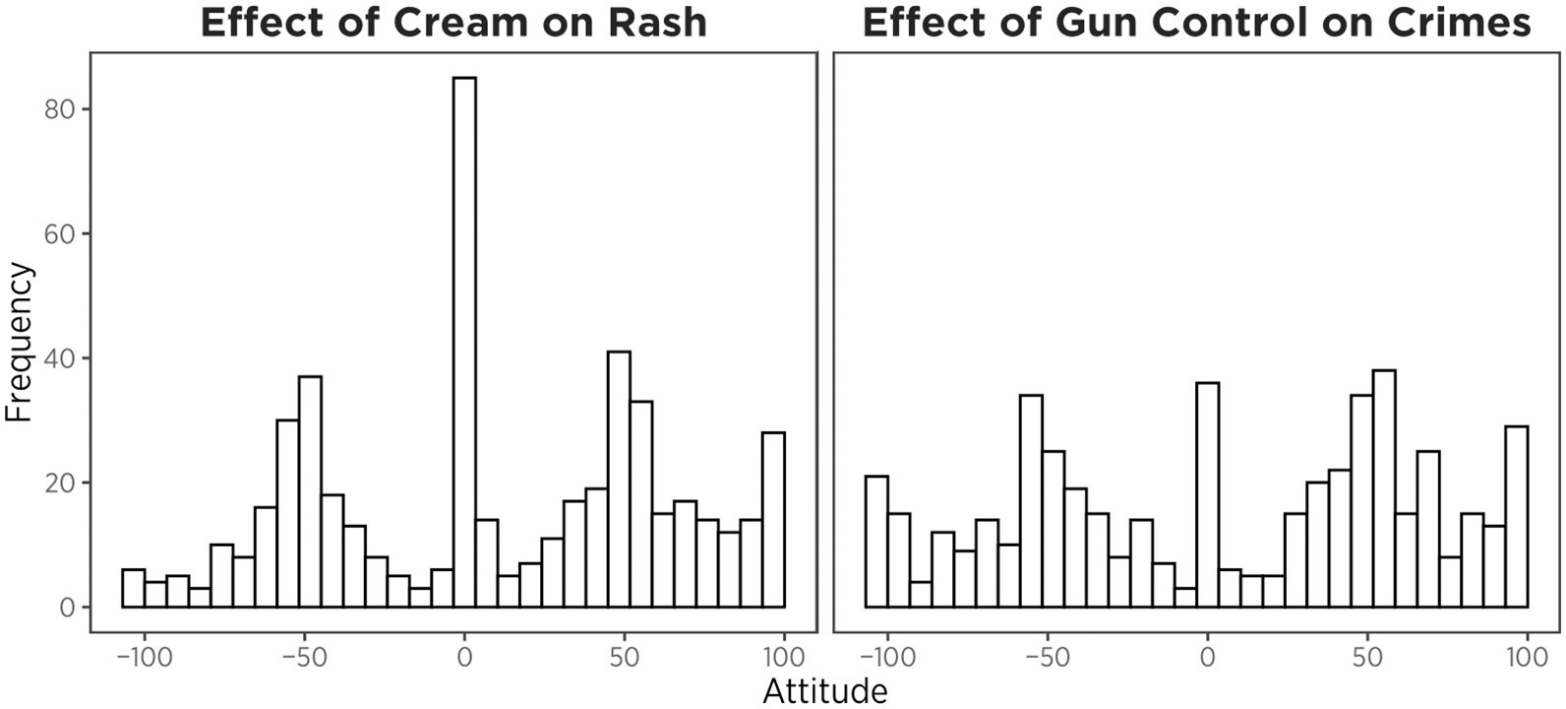
Frequency of prior attitude towards the effect of cream on rash and effect of gun control on crimes. -100 means strongly likely to decrease, 0 means equally likely to decrease or increase, and 100 means strongly likely to increase.

Before completing the covariance task, participants’ attitudes on the issue to which they were randomly assigned (crime/rash) was measured. We expected (and wished to confirm) that political outlooks were related to attitudes about crime control not rash control. Participants were asked ‘What is your opinion on whether gun control (a new skin cream) is likely to decrease or increase crime (rash)?’, and they needed to rate on a continuous bar with labels ‘Strongly likely to decrease crime (rash)’, coded as -100, ‘Somewhat likely to decrease crime (rash)’, coded as -50, ‘Equally likely to decrease or increase crime (rash)’, coded as 0, ‘Somewhat likely to increase crime (rash)’, coded as 50, and ‘Strongly likely to increase crime (rash)’, coded as 100. Participants’ rates could be anywhere on this spectrum (see Fig 2 for the distribution).

Among those who received the crime question (*N* = 496), attitudes were not significantly different from the neutral standing that it is equally likely to decrease or increase crime (*Mean* = 4.78, *SD* = 60.64, *t* = 1.75, *df* = 495, *p* = .080). Among those who received the rash question (*N* = 504), participants attitudes slightly leaned towards the claim that a new skin cream would likely increase rash (*Mean* = 9.45, *SD* = 53.73, *t* = 4.07, *df* = 503, *p* < .001).

#### Political outlook

We used the two-item political outlook measures from Kahan et al. (2017)’s study [3], including political party identification and political ideology. Participants’ political party identification was measured by a 7-point Likert scale (‘Strong Democrat’, ‘Democrat’, ‘Independent Lean Democrat’, ‘Independent’, ‘Independent Lean Republican’, ‘Republican’, and ‘Strong Republican’). Participants’ political ideology was measured by a 5-point Likert scale (‘Very Liberal’, ‘Liberal’, ‘Moderate’, ‘Conservative’, and ‘Very Conservative’). Political party identification and political ideology were moderately correlated (*r* = .50, *p* < .001). Internal reliability was moderate (Cronbach’s *alpha* = .66). For better interpretation, participants’ responses to these two items were aggregated and standardized as their scores for Political Orientation [3]. A higher value of Political Orientation means a stronger tendency towards Conservative/Republican.

Somewhat surprisingly, Political Orientation was only weakly associated with the attitude that gun control increases crime (*r* = .24, *N* = 496, *p* < .001), indicating that people who lean towards conservative Republicans were more likely to believe that gun control increases crime. As expected, political orientation was unrelated to attitudes regarding a new skin cream increasing a rash (*r* = .03, *N* = 504, *p* = .39).

#### Numeracy scale

**Fig 3:**
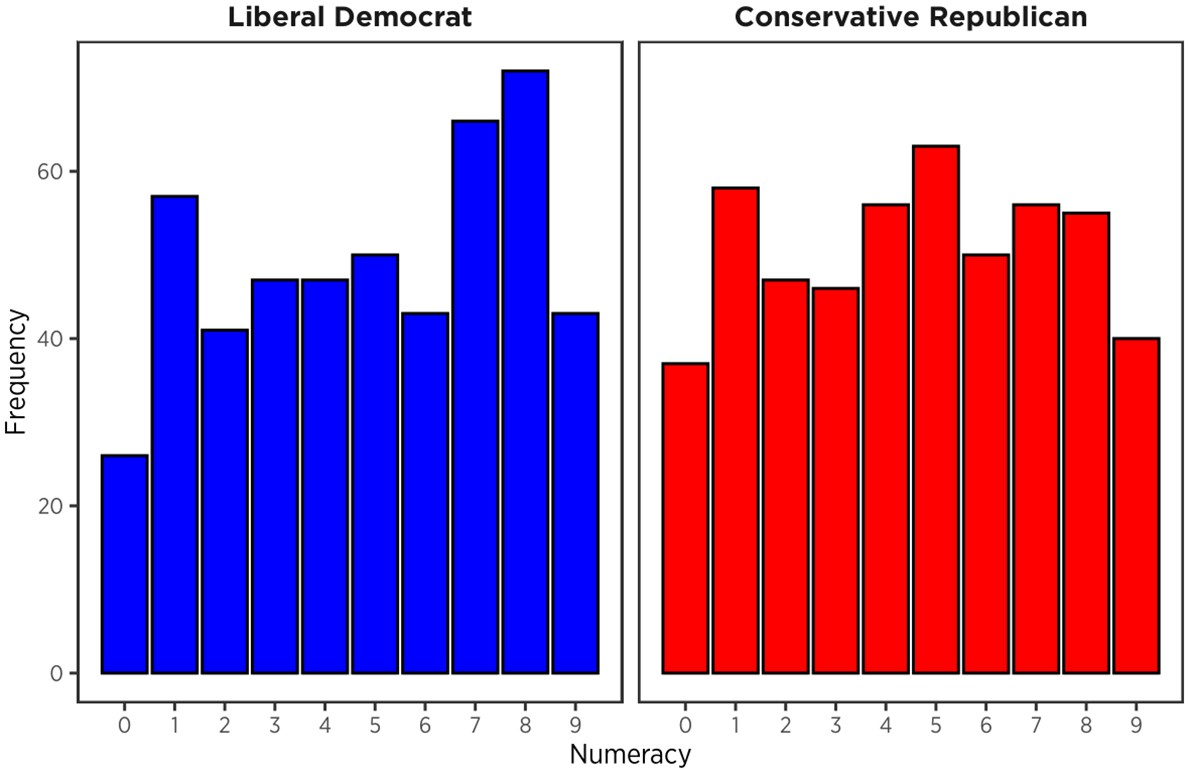
Frequency of numeracy scores. Participants with standardized Political Orientation < 0 were coded as ‘Liberal Democrat’ and Political Orientation > 0 as ‘Conservative Republican’.

We used the 9-item numeracy scale used in Kahan et al. (2017)’s study [3]. It consisted of 8 items from the Abbreviated Numeracy Scale [63], which conventionally measures quantitative reasoning. In addition, an additional item from the Cognitive Reflective Task (CRT) [64] was included, because CRT measures the ability to overcome heuristic beliefs, and performance on CRT reliably predicts performance on heuristic and biases tasks [65]. Mean number of correct responses was 4.72 (*SD* = 2.77). Internal reliability was high (Cronbach’s *alpha* = .84). Numeracy skill did not differ among those who scored above and below the mean on Political Orientation, (*t* = -1.78, *df* = 996, *p* = .076, see Fig 3 for the distribution).

## Results

Because the second trial of the covariance task presented the same data as the first trial in another format, the accuracy of the second presentation is heavily biased by the first presentation. Therefore, to examine motivated numeracy and the effect of presentation format (i.e., pre-registered hypotheses 1-4 and exploratory analyses), only the first trial was included in the analyses. Accuracy for the second trial was analyzed to examine the transfer effect of number line presentation (i.e., pre-registered hypothesis 5).

### Pre-registered analyses

Based on our preregistered analysis plan, we fit a logistic regression model with the correct interpretation of data (=1) of the first trial of the covariance task as the dependent variable, and with mean-centered numeracy, task type (crime increases, crime decreases, rash increases, and rash decreases), mean-centered Political Orientation, format (table, line), and all interactions as predictors. Odds ratios of different stages of the model are reported in Table 1.

**Table 1:** Odds ratios for the logistic regression model.

Predictors	Stage 1	Stage 2	Stage 3	Stage 4
Intercept	1.52 **	1.59 *	1.63 *	1.65 *
Numeracy	1.14 *	1.14 *	0.69 **	0.55 **
Rash_decreases	0.84	0.81	0.74	0.72
Rash_increases	0.26 ***	0.25 ***	0.18 ***	0.15 ***
Crime_increases	0.27 ***	0.24 ***	0.21 ***	0.21 ***
Political Orientation	0.90	0.90	0.66 **	0.60 *
Format	1.75 ***	1.59	1.57	1.54
Format * Rash_decreases		1.11	1.27	1.25
Format * Rash_increases		1.06	1.10	1.47
Format * Crime_increases		1.27	1.33	1.41
Numeracy * Rash_decreases			0.91	0.91
Numeracy * Rash_increases			4.68 ***	8.56 ***
Numeracy * Crime_increases			2.21 ***	2.42 **
Numeracy * Political Orientation			0.87	0.71
Political Orientation * Rash_decreases			1.16	1.04
Political Orientation * Rash_increases			1.47	1.89
Political Orientation * Crime_increases			1.98 ***	2.28 **
Numeracy * Rash_decreases * Political Orientation			1.12	1.40
Numeracy * Rash_increases * Political Orientation			1.23	1.15
Numeracy * Crime_increases * Political Orientation			0.79	0.79
Format * Political Orientation				1.27
Format * Numeracy				1.50
Format * Political Orientation * Numeracy				1.43
Format * Political Orientation * Rash_decreases				1.20
Format * Political Orientation * Rash_increases				0.63
Format * Political Orientation * Crime_increases				0.71
Format * Numeracy * Rash_decreases				1.00
Format * Numeracy * Rash_increases				0.38 *
Format * Numeracy * Crime_increases				0.83
Format * Numeracy * Political Orientation * Rash_decreases				0.64
Format * Numeracy * Political Orientation * Rash_increases				1.03
Format * Numeracy * Political Orientation * Crime_increases				0.98
F-test	F(6, 993) = 111.02 ***	F(9, 990) = 111.45 ***	F(19, 980) = 212.36 ***	F(31, 968) = 226.09 ***
Delta F-test		F(3, 990) = 0.43	F(10, 980) = 100.91 ***	F(12, 968) = 13.73
BIC	1323.62	1343.92	1312.09	1381.25

*Note*: The reference group for Task Type is crime decreases. The reference group for Format is table. Numeracy is mean-centered. Political Orientation: positive values indicate more conservative/Republican orientation and negative values indicate more liberal/Democrat orientation.

*** *p* < .001,

** *p* < .01,

* *p* < .05.

#### Pre-registered Hypothesis 1: Number-lines improve accuracy

We hypothesized that people would be more accurate interpreting data presented in the number-line than table format (Hypothesis 1), indicated by a main effect of format. As expected, accuracy with the number-line format was higher than the table format (56% vs 43%, *χ*^2^(1) = 17.66, *p* < .001). The odds of correctly solving the covariance problem presented in the line format increased by 75% than problems presented in the table format (OR = 1.75, 95% CI [1.35, 2.28], *p* < .001).

#### Pre-registered Hypothesis 2: Number-lines improve accuracy by reducing bias

We expected that number-lines improve accuracy by reducing bias (Hypothesis 2) indicated by a two-way interaction between problem type and format. Inconsistent with our expectation, the interaction effect between problem type and format was not significant (*χ*^2^(3) = 0.43, *p* = .934), indicating that number lines did not help more on political problems than non-political problems.

#### Pre-registered Hypothesis 3: Numeracy amplifies political bias (“Motivated numeracy”)

We hypothesized that numeracy amplifies political bias (“Motivated numeracy”, Hypothesis 3), indicated by a significant three-way interaction among problem type, political outlook, and numeracy. Contrary to our hypothesis, the three-way interaction among problem type, political outlook, and numeracy was not significant (*χ*^2^(3) = 4.27, *p* = .234), indicating a lack of motivated numeracy.

#### Pre-registered Hypothesis 4: Number-lines improve accuracy by reducing motivated numeracy (“Cognitive support for objective numeracy”)

We hypothesized that number-lines improve accuracy by reducing motivated numeracy (Hypothesis 4), indicated by a significant four-way interaction among problem type, format, political outlook, and numeracy. Contrary to our hypothesis, we did not observe a significant four-way interaction among problem type, format, political outlook, and numeracy (*χ*^2^(3) = 1.58, *p* = .663).

#### Pre-registered Hypothesis 5: The transfer effect of number line

Based on our preregistered analysis plan, we conducted a logistic regression model with accuracy of solving the covariance problems (both the first and the second trial) as the dependent variable, and with trial number (1st, 2nd), mean-centered numeracy, problem type (crime increases, crime decreases, rash increases, and rash decreases), mean-centered Political Orientation, format (table, line), and all interactions as predictors.

**Fig 4:**
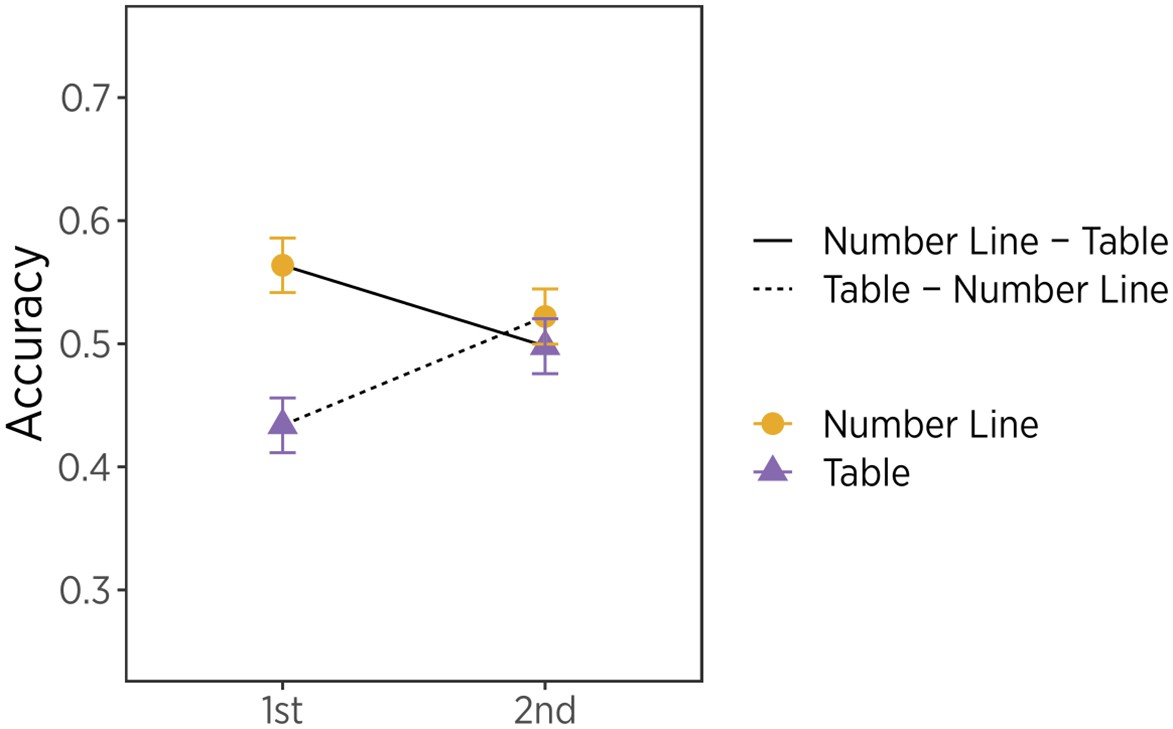
Average accuracy across formats and trial number. Error bars indicate standard errors.

Results showed a format by trial number interaction (*χ*^2^(1) = 5.06, *p* < .05, Fig 4). The nominal accuracy of interpreting data in the table format after line format (*Mean* = .50, *SD* = .50) was slightly higher than table format first (*Mean* = .43, *SD* = .50), whereas accuracy of interpreting data in line format after table format (*Mean* = .52, *SD* = .50) was slightly lower than the line format first (*Mean* = .56, *SD* = .50). Statistically, accuracy of solving table problems after line problems was reliably higher than accuracy of table solutions first, t(998) = 2.04, *p* < .05. These results held after controlling for other variables (e.g., numeracy skills, task type, and political outlook), OR = 1.30, 95% CI [1.00, 1.68], *p* < .05. Results suggest that presenting data in the line format has a positive transfer effect on the following problem. Further testing of line-then-line and table-then-table conditions is necessary to test this idea.

To summarize, our results failed to replicate “motivated numeracy”. However, we observed an advantage of the number-line format over the table format across politically controversial and neutral questions. More importantly, presenting data in the line format increased the probability of correctly interpreting data in the data format in the following question, suggesting a promising intervention.

### Exploratory analyses

To further understand the non-replication of motivated numeracy, we conducted three sets of unplanned analyses: 1) Bayesian analyses for motivated numeracy, 2) a comparison of effect sizes of our study to previous studies that failed to replicate motivated numeracy, and 3) investigating the effect of prior factual belief on numerical reasoning.

#### Bayesian analyses for motivated numeracy

We conducted a Bayesian logistic regression analysis to better interpret the non-significant results of our pre-registered hypothesis 3, i.e., numeracy amplifies political bias (“motivated numeracy”). Bayesian analyses allow us to gain additional insights regarding the degrees of evidence favoring the null hypothesis [66]. We used a multivariate Cauchy distribution [67] as priors on the coefficients, and a uniform distribution for prior model probabilities. The analyses was conducted in JASP 0.19.0 [68].

For the purpose of comparison, we ran the same pre-registered regression model (Table 1, step 3) using Bayesian analyses. Posterior summaries for each of the regression coefficients were reported in Table 2. The odds for including the three-way interaction among problem type, political outlook, and numeracy have increased by a factor of 1.66, indicating only anecdotal evidence for motivated numeracy. Therefore, we did not observe sufficient evidence supporting motivated numeracy.

**Table 2:** Posterior summaries of coefficients in Bayesian logistic regression.

Coefficients	*M* (*SD*)	95% CI	BF_*incl*_
Intercept	0.42 (.16)	[0.11, 0.72]	1.00
Numeracy	-0.35 (.13)	[-0.60, -0.09]	9.71 * 10^14^
Rash_decreases	-0.18 (.19)	[-0.53, 0.20]	1.24 * 10^31^
Rash_increases	-1.51 (.21)	[-1.91, -1.10]	1.24 * 10^31^
Crime_increases	-1.26 (.19)	[-1.61, -0.86]	1.24 * 10^31^
Political Orientation	-0.37 (.16)	[-0.62, 0.00]	16.43
Format	0.55 (.15)	[0.29, 0.84]	1310.15
Format * Rash_decreases	0.01 (.08)	[0.00, 0.00]	0.09
Format * Rash_increases	0.01 (.07)	[0.00, 0.00]	0.09
Format * Crime_increases	0.01 (.08)	[0.00, 0.00]	0.09
Numeracy * Rash_decreases	-0.08 (.19)	[-0.45, 0.29]	9.09 * 10^14^
Numeracy * Rash_increases	1.41 (.22)	[1.00, 1.84]	9.09 * 10^14^
Numeracy * Crime_increases	0.71 (.19)	[0.34, 1.07]	9.09 * 10^14^
Numeracy * Political Orientation	-0.07 (.09)	[-0.26, 0.01]	2.56
Political Orientation * Rash_decreases	0.14 (.18)	[-0.19, 0.50]	29.44
Political Orientation * Rash_increases	0.39 (.22)	[-0.02, 0.75]	29.44
Political Orientation * Crime_increases	0.60 (.24)	[0.00, 0.94]	29.44
Numeracy * Rash_decreases * Political Orientation	0.01 (.07)	[-0.09, 0.17]	1.66
Numeracy * Rash_increases * Political Orientation	0.02 (.09)	[-0.05, 0.27]	1.66
Numeracy * Crime_increases * Political Orientation	-0.02 (.09)	[-0.23, 0.02]	1.66

*Note*: The reference group for Task Type is crime decreases. The reference group for Format is table. Numeracy is mean-centered. Political Orientation: positive values indicate more conservative/Republican orientation and negative values indicate more liberal/Democrat orientation. *BF*_*incl*_, the inclusion Bayes factor, i.e., the Bayes factor by which the data have increased the prior odds for including a covariate. CI, credible interval. *** *p* < .001, ** *p* < .01, * *p* < .05.

#### Comparison to previous failures of replications of motivated numeracy

Our study is not the first failure of replication of the “motivated numeracy” [26, 30, 33, 69–71]. For example, Persson et al. (2021) conducted a large-scale pre-registered replication of “motivated numeracy” with 3000 participants and failed to observe a general interaction between political ideology and numeracy [33]. For the purpose of comparison, we conducted the same regression analyses as in Persson et al. (2021). More specially, we conducted separate simple logistics regression models on the accuracy of solving the covariance problems on the first trial for the four task types, (i.e., crime increases, crime decreases, rash increases, and rash decreases) respectively, with mean-centered numeracy, political outlook, and format as predictors (Table 3), and full logistics regression models with the interaction effect between numeracy and political outlook and the interaction effect between numeracy and format (Table 4). Consistent with Persson et al. (2021), we did not observe a consistent motivated numeracy effect, indicated by a significant interaction between numeracy and political orientation (crime increases: OR = 0.69, 95% CI = [.51, .92], *p* < .05; crime decreases: OR = 0.85, 95% CI = [.64, 1.13], *p* = .278).

**Table 3:** Odds ratios for the logistic regression model.

	Crime increases	Crime decreases	Rash increases	Rash decreases
Numeracy	1.48 **	0.68 **	3.19 ***	0.62 **
Political Orientation	1.32	0.65 **	1.00	0.76 *
Format	2.09 **	1.59	1.73	1.99 *
Constant	0.36 ***	1.64 *	0.30 ***	1.21
N	247	249	251	253

*Note*: Dependent variable is the correct interpretation of data (=1). Numeracy is mean-centered. Political Orientation is a mean-centered composite measure of two underlying variables measuring party identification and political ideology, with positive values denoting Republican/conservative, and negative values denoting Democrat/liberal. The reference group for Format was table.

* *p* < 0.05;

** *p* < 0.01;

*** *p* < 0.001.

**Table 4:** Odds ratios for the logistic regression model.

	Crime increases	Crime decreases	Rash increases	Rash decreases
Numeracy	1.30	0.57 **	4.53 ***	0.51 **
Political Orientation	1.29	0.66 **	0.99	0.76 *
Format	2.02 *	1.54	2.23 *	1.95 *
Numeracy * Political Orientation	0.69 *	0.85	1.04	0.97
Numeracy * Format	1.31	1.49	0.57	1.44
Constant	.36 ***	1.64 *	0.25 ***	1.19
N	247	249	251	253

*Note*: The dependent variable is the correct interpretation of data (=1). Numeracy is mean-centered. Political Orientation is a mean-centered composite measure of two underlying variables measuring party identification and political ideology, with positive values denoting Republican/conservative, and negative values denoting Democrat/liberal. The reference group for Format was table.

* *p* < 0.05;

** *p* < 0.01;

*** *p* < 0.001.

#### Attitude as motivation

Although one’s party identification and political ideology predict attitude toward policy-relevant facts, individual prior factual belief can also have an effect on numerical reasoning. If heuristic belief, rather than political group identity, serves as the underlying mechanism of “motivated numeracy”, then we would expect that 1) prior attitudes serve as a stronger predictor than political outlook, and 2) prior attitudes have an effect on reasoning about politically neutral problems more generally (e.g., the rash problems).

In fact, we observed that political outlook was only weakly associated with the belief that gun control increases crime (*r* = .24, *N* = 496, *p* < .001), suggesting that the failure to replicate “motivated numeracy” might be due to the fact that political outlook is not a reliable proxy for gun control attitudes. Further, somewhat surprisingly, among those who received the rash question (*N* = 504), 56.94% of participants’ prior attitudes leaned toward the claim that a new skin cream would be likely to increase rashes, and 38.10% of participants’ prior attitudes leaned toward the opposite, suggesting that the neutral rash problem was not exactly neutral. These findings suggested that actual prior attitudes on gun control and rash treatments provided a more robust test of motivated numeracy than political affiliation.

To test the effect of prior attitude on the accuracy of data interpretation, we computed attitude consistency as how strongly participants’ prior attitude was consistent with what the data interpretation problem conveyed. Specifically, attitude consistency was coded as attitude (i.e., how strongly participants agreed with “cream/gun control would increase rash/crime”) when the true interpretation of data conveyed that cream/gun control would increase rash/crime, and was coded as reversed attitude when the true interpretation of data conveyed the opposite. Thus, attitude consistency is a continuous variable ranging from -100 to 100. Larger attitude consistency means that the participant more strongly agreed with what the data conveyed.

We conducted a logistic regression model with the accuracy of solving the covariance task on the first trial as the dependent variable, and with mean-centered numeracy, mean-centered attitude consistency, format (table, line), task (crime, rash), and all interactions as predictors. Table 5 reported odds ratios of model predictors across different stages of the model (Stage 1 model only included main effects; Stage 2 model added two-way interactions to Stage 1 model; Stage 3 model added three-way interactions to Stage 2 model; Stage 4 added four-way interactions to Stage 3 model).

**Table 5:** Odds ratios for the logistic regression model.

Predictors	Stage 1	Stage 2	Stage 3	Stage 4
Intercept	0.79 *	0.78	0.78	0.78
Numeracy	1.12	0.91	0.81	0.81
Task	0.93	0.86	0.80	0.78
Format	1.70 ***	1.75 **	1.76 **	1.75 **
Attitude	0.75 ***	0.91	0.98	0.97
Task * Numeracy		1.37 *	1.86 **	1.91 **
Task * Format		0.99	1.09	1.13
Task * Attitude		0.48 ***	0.38 ***	0.38 ***
Format * Attitude		1.42 *	1.23	1.24
Format * Numeracy		1.17	1.43	1.42
Numeracy * Attitude		1.63 ***	1.74 ***	1.64***
Numeracy * Task * Format			0.63	0.59
Numeracy * Task * Attitude			1.11	1.34
Task * Format * Attitude			1.48	1.47
Format * Attitude * Numeracy			0.84	0.94
Numeracy * Task * Format * Attitude				0.73
F-test	F(4, 995) = 39.93 ***	F(10, 989) = 134.81 ***	F(14, 985) = 140.39 ***	F(15, 984) = 141.43***
Delta F-test		F(6, 989) = 94.88 ***	F(4, 985) = 5.57	F(1, 984) = 1.04
BIC	1380.90	1327.46	1349.52	1355.39

*Note*: Numeracy and Attitude was mean-centered. A larger Attitude means that participants more strongly agree with what the data conveys. The reference group for Format was Table. The reference group for Task was crime.

* *p* < 0.05;

** *p* < 0.01;

*** *p* < 0.001.

We found that the odds of correctly interpreting data presented in the line format increased by 70% compared to data presented in the table format (OR = 1.70, 95% CI = [1.32, 2.19], *p* < .01, Table 5). We also found that there was an interaction between task and attitude (*χ*^2^(1) = 26.51, *p* < .001). The accuracy of interpreting crime questions was higher than the rash questions when data affirmed participants’ attitudes (*Mean* = .50 vs. .37). In contrast, accuracy of crime solutions was lower than rash solutions when data disaffirmed participants’ attitudes (*Mean* = .52 vs. .61). Additionally, there was also an interaction between task and numeracy (*χ*^2^(1) = 4.99, *p* < .05). Numeracy skills more strongly predicted accuracy for rash problems compared to crime problems.

**Fig 5:**
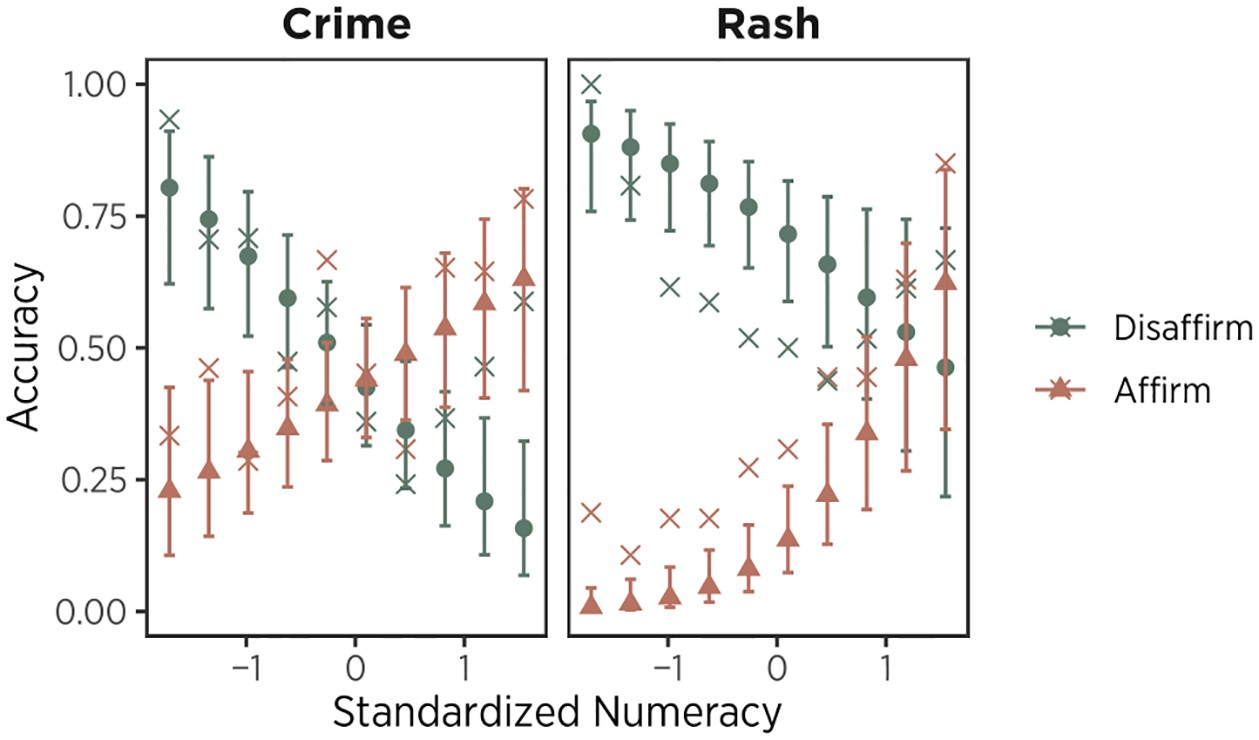
Model predicted accuracy by standardized numeracy and attitudes. Predictors for Attitude were set at -1.5 SD and +1.5 SD for ‘Disaffirm’ and ‘Affirm’, respectively. Error bars indicate 95% confidence intervals. Crosses indicate raw average accuracy.

Most importantly, there was an interaction between numeracy and attitude (*χ*^2^(1) = 49.19, *p* < .001, Fig 5). The accuracy of interpreting affirming conclusions increased as numeracy skills increased, whereas the accuracy of interpreting disaffirming conclusions decreased as numeracy skills decreased. Thus, data did indicate ‘motivated numeracy’, but motivation was largely driven by prior beliefs rather than political affiliation per se.

We did not observe a three-way interaction effect among numeracy, attitude, and task (*χ*^2^(1) = .45, *p* = .503), suggesting that participants demonstrated ‘motivated numeracy’ in both rash and crime problems. This is also consistent with the idea that motivated numeracy is about preserving prior beliefs rather than preserving political ideology. Indeed, separate models on rash and crime tasks showed that the effect of “motivated numeracy” (i.e., the interaction between numeracy and attitude) was significant in both rash and crime problems (rash: OR = 2.10, 95% CI = [1.46, 3.12], *p* < .001; crime: OR = 1.69, 95% CI = [1.29, 2.26], *p* < .001, full model reported in Table 6).

**Table 6:** Odds ratios for the logistic regression model.

	Crime	Rash
Intercept	0.78	0.59 **
Numeracy	0.80	1.59 **
Attitude	0.96	0.39 ***
Format	1.73 **	2.01 ***
Format * Attitude	1.26	1.75 *
Format * Numeracy	1.43	0.83
Numeracy * Attitude	1.69 ***	2.10 ***
Format * Attitude * Numeracy	0.94	0.70
F-test	F(7, 488) = 41.66 ***	F(7, 496) = 99.28 ***

*Note*: Numeracy was mean-centered. Attitude was mean-centered. Larger attitude consistency means the participant more strongly agreed with what the data conveyed. The reference group for Format was Table.

* *p* < 0.05;

** *p* < 0.01;

*** *p* < 0.001.

Again, we also did not observe a three-way interaction effect among numeracy, attitude, and format (*χ*^2^(1) = 1.41, *p* = .235). This indicated that the line format yielded more accurate data interpretation than the table format, regardless of whether or not the data affirmed participants’ prior attitudes. However, the line format also did not reduce bias. Separate logistical regression models on the table and number line format showed that the effect of “motivated numeracy” was significant in both formats (number line: OR = 1.57, CI [1.22, 2.06], *p* < .001; table: OR = 1.61, CI [1.26, 2.09], *p* < .001, Table 7). No four-way interactions were found to be significant.

**Table 7:** Odds ratios for the logistic regression model.

	Number line	Table
Intercept	1.37 *	0.78
Numeracy	1.17	0.82
Attitude	1.23	0.96
Task	0.88	0.77
Task * Attitude	0.55 **	0.38 ***
Task * Numeracy	1.12	1.87 **
Numeracy * Attitude	1.57 ***	1.61 ***
Task * Attitude * Numeracy	0.98	1.33
F-test	F(7, 494) = 42.39 ***	F(7, 490) = 82.09 ***

*Note*: Numeracy was mean-centered. Attitude was mean-centered. Larger attitude consistency means the participant more strongly agreed with what the data conveyed. The reference group for Task was crime.

* *p* < 0.05;

** *p* < 0.01;

*** *p* < 0.001.

In summary, “motivated numeracy” was observed when we used prior attitude, rather than “political outlook”, as an indicator of motivation. The effect of “motivated numeracy” was similar for the politically controversial and politically neutral questions. Again, we observed an effect of format. Presenting data in the line format increased accuracy compared to presenting data in the table format. However, the number-line format did not reduce “motivated numeracy” itself.

## Discussion

We hypothesized that cognitive supports that help children’s proportional reasoning would also help political partisans’ numerical objectivity. Consistent with this hypothesis, we observed that data presented in number-line formats yielded greater accuracy than table formats. Number-lines improved accuracy when the true interpretation of data affirmed, was neutral to, or disaffirmed participants’ political outlooks. Moreover, solving table problems after number-line problems yielded greater accuracy than solving table problems first, which suggests that number-line practice may have been educational.

Against predictions, however, number lines did not eliminate the confirmation bias altogether. With the number-line format, even participants with high numeracy skills were more likely to correctly interpret self-confirming data than disconfirming data. In other words, motivated reasoning was found even with this simplified presentation format, providing further evidence that motivated reasoning is hard to overcome [5]. This is also consistent with findings that providing repeated evidence might have little impact on individuals’ attitude roots, such as ideology, values, worldview, and so on [72].

Our study holds promising educational implications with the potential to address common misinterpretations of rational numbers. Although misinterpretation of rational numbers is common, they are malleable and can be improved with intervention [21, 23, 59, 60]. Our number-line approach showed that effective strategies of data presentation can enhance quantitative skills. This approach suggests broader applications in science communication, especially in contexts like health decision-making. Health-related statistics often involve rational numbers, such as the lifetime risk of breast cancer or mortality rate of COVID-19. Our number line approach can be readily adapted to improve the interpretation of medical data, helping individuals make more informed healthcare decisions, and better understand public health policies.

Beyond improving reasoning, another purpose of this study was to replicate the “motivated numeracy” effect. On the one hand, we did not observe “motivated numeracy” [3] based on political identity, which was inconsistent with some successful replications [25]. On the other hand, “motivated numeracy” was observed based on prior beliefs about gun control. This finding is consistent with Tappin et al. (2020) [73], which demonstrated that prior beliefs matter more than political identity in motivated reasoning.

Previous studies have reached different conclusions about motivated numeracy [25, 27]. In many previous studies, the root of the motivation is based on political ideology generally [26, 33, 69, 70]. However, a more general hypothesis is that motivated numeracy is primarily driven by specific prior beliefs, and the extent to which prior beliefs correlate with political identity will determine the extent to which political identity affects motivated numeracy. A graded correlation between political identity and prior beliefs is already present somewhat within existing studies, with a non-zero correlation for beliefs about gun control and political identity and a near-zero correlation for beliefs about skin creams and political identity. Between studies, however, the correlation between beliefs about gun control and political identity varies.

An implication of these findings is that the probability of a replication depends on how well the political outlook matches gun control attitudes, which may change over time and locale. Indeed, the engagement of motivated numeracy varies across scenarios. For example, participants with higher numeracy skills were less likely to engage in motivated reasoning when interpreting data on the effect of refugee intake on crime rates, but were more likely to engage in motivated reasoning when interpreting data on the effect of gender quotas on companies’ financial results [71]. On the other hand, recent studies have also found no evidence for motivated numeracy based on prior factual beliefs about multiple controversial scenarios [27, 28]. Additionally, note that attitude roots that drive motivated reasoning can be unconscious and hidden under surface attitudes [72]. A specific surface attitude can be supported by multiple attitude roots, which are likely to be different for different individuals and might change over time. A further meta-analysis is warranted to examine the possibility that we suggest.

In evaluating the motivated numeracy effect, we also observed a complication arising from the pattern of responses for the rash problem. Although the rash problem has been used in previous studies as a neutral reference condition [3, 16, 33], attitudes on the rash problem were similar in some ways to the crime problem. Like the crime problem, prior beliefs about the rash were not distributed normally (see Fig 2). Rather, like the crime problem, non-neutral prior attitudes tended to be bimodal, with the distance between the two modal responses—a conventional measure of polarization—being similar for rash and crime problems. Thus, when confronting data about the effects of cream/gun control, most participants were confronting data that affirmed or disaffirmed a prior attitude. We also observed a “motivated numeracy” effect in rash problems based on prior attitude in our exploratory analyses. Perhaps a simple way to understand ‘motivated numeracy’ is that it reflects general over-confidence among the numerically adept.

Future studies on motivated numeracy would be improved by measuring attitudes both before and after exposure to data. This approach addresses whether politically related attitudes are less malleable than those associated with politically neutral questions. Using multiple politically controversial and neutral questions would also better address issues regarding polarization. As political scientists have noted [1], the alignment of attitudes on multiple issues may pose a unique threat to attitude change, and the alignment of attitudes likely provides a more robust test of partisanship than political identity.

Although the relationship between attitude towards gun control and political partisanship is a culturally specific issue within the United States, our findings hold the potential for generalization across different societies and cultures. For example, motivated reasoning has been demonstrated in controversial topics that are less directly related to political partisanship such as capital punishment, pacifism, and nanotechnology [74–76], providing a rich context to explore how numeracy skills and information presentation format moderate motivated numeracy across cultures. Consequently, “motivated numeracy” has indeed been extended beyond the US, using culturally general topics. For example, “motivated numeracy” was observed when political partisanship motivated Australian participants to assess climate change risks in a biased manner [25]. Additionally, worldview ideologies and values also serve as motivations beyond political party identification. For instance, nationally versus globally oriented ideologies biased Swedish participants’ interpretation of immigration policy data, with numeracy skills exaggerating the polarization [71]. Therefore, individuals’ opinions on controversial issues might serve as more general mechanisms underlying “motivated numeracy” than partisanship-biased attitudes alone. Measuring prior attitudes towards controversial issues can help generalize findings to different cultures.

We should note that one limitation of our study is that our 1000 participants were drawn from MTurk. MTurk samples may skew more Liberal and Democrat than the general US population [77], suggesting that future replications should recruit a more representative sample. On the other hand, others have found that convenience samples such as those recruited from MTurk yielded at least as reliable results as those from representative samples [78–80].

Another limitation regards the transfer effect of number line training. To test whether completing number line questions would lead to better accuracy without cognitive resources of number lines, we directly compared the accuracy of the table condition after the line condition and the accuracy of completing the table condition first. Without a full 2 (format) by 2 (trial number) design, it is hard to distinguish the effect of transfer of learning versus the effect of practice. Further testing of line-then-line and table-then-table is needed to more rigorously test this prediction.

In summary, we aimed to use number lines to help political partisans interpret data more objectively, as opposed to only using their numerical skills to affirm their pre-existing beliefs. As expected, the number line did increase accuracy, although it did not reduce “motivated numeracy”, which may be a general over-confidence among the numerical adept. Our findings suggest that if scientists wish to improve understanding of politically sensitive statistics, presenting data in number-line format is likely to be beneficial.
